# Bibliometric and visualized analysis of sodium–Glucose cotransporter 2 inhibitors

**DOI:** 10.3389/fphar.2022.1009025

**Published:** 2023-01-04

**Authors:** He Sun, Zhongqing Wang, Yuxi Wang, Haichuan Rong, Danyang Wang, Xiangnian Liu, Ke Jin, Zhicheng Sun, Qiuling Fan

**Affiliations:** ^1^ Department of Nephrology, The First Hospital of China Medical University, Shenyang, China; ^2^ Department of Endocrinology, Shengjing Hospital of China Medical University, Shenyang, China; ^3^ Department of Information Center, The First Hospital of China Medical University, Shenyang, China; ^4^ Department of Clinical Medicine, China Medical University, Shenyang, China; ^5^ Department of Nephrology, Shanghai General Hospital, Shanghai Jiao Tong University School of Medicine, Shenyang, China

**Keywords:** sodium—glucose cotransporter 2 inhibitor, bibliometric, visualization, heart failure, diabetic cardiomyopathy, co-occurrence analysis

## Abstract

**Background:** Sodium—glucose cotransporter 2 inhibitors have proved to be extremely effective and reliable in reducing hyperglycemia, and have also been used for the treatment of cardiovascular and renal disease in patients with or without type 2 diabetes. Thousands of research articles on SGLT2 inhibitors have been published in the past, but few bibliometric analyses have systematically been studied this field. We aimed to visualize the global research hotspots and trends of SGLT2 inhibitors using a bibliometric analysis to provide new evidence and ideas for researchers and clinicians in this field.

**Methods:** We retrieved publications from Science Citation Index Expanded of Web of Science Core Collection in 2004–2022 on 1 July 2022. Microsoft Excel, CiteSpace and VOSviewer were employed to collect publication data, analyze publication trends, and visualize relevant results.

**Results:** We identified 4,419 original research articles on SGLT2 inhibitors published between 2004 and the first half of 2022. Global SGLT2 inhibitors-related research increased rapidly from 2004 to 2022, especially recently. United States made the greatest contribution to the topic, with (1,629, 36.86%) publications and citations (88,892). AstraZeneca was the most prolific institutions (272, 6.16%). Heerspink HJL published the most related articles (98), whereas Zinman B was cited the most frequently (1,784 citations). Diabetes Obesity and Metabolism was the journal with the most studies (406, 9.19%), and The New England Journal of Medicine was the most commonly cited journal (11,617 citations), with nine of the top 10 co-cited references published in this journal. The emerging keywords “heart failure,” “diabetic cardiomyopathy,” “ejection fraction,” “mortality,” “biomarker,” “fibrosis,” “ampk,” and “guideline” appeared the most recently as research frontiers.

**Conclusion:** United States is the leader in SGLT2 inhibitor research. Recently, the research on SGLT2 inhibitors has focused on clinical trials, related mechanisms, and therapy. In the future, the research on SGLT2 inhibitors will delve into molecular mechanisms, especially those related to fibrosis and AMPK, revealing the link between SGLT2 inhibitors and heart failure and diabetic cardiomyopathy will be the next research hotspot.

## 1 Introduction

Sodium–glucose cotransporter 2 (SGLT2) inhibitors, a new class of oral hypoglycemic agents, ameliorate hyperglycemia by inhibiting glucose reabsorption in the proximal tubule of the kidney, thereby functioning in an insulin-independent manner (DeFronzo et al., 2017). In addition, SGLT2 inhibitors improve glucose tolerance by reducing both the threshold for glucosuria and the maximum glucose resorptive capacity and by ameliorating glucotoxicity, leading to enhanced β-cell function and improved insulin sensitivity in muscles (Bertinat et al., 2016; Goto et al., 2020). SGLT2 inhibitors have confirmed hypoglycemic effects with no risk of hypoglycemia; they can also reduce body weight and blood pressure, increase natriuresis and diuresis, and afford additional cardiovascular and renal protection (Bolinder et al., 2014; Neeland et al., 2016; Park et al., 2020). Notably, SGLT2 inhibitors can reduce hospitalization for heart failure and delay the progression of renal disease, regardless of existing atherosclerotic cardiovascular disease or heart failure history (Fitchett et al., 2016; Neal et al., 2017; Wiviott et al., 2019; Zelniker et al., 2019). Compared with traditional hypoglycemic drugs, SGLT2 inhibitors may bring greater therapeutic benefits to patients with diabetes. Currently, several SGLT2 inhibitors are already available in many countries (dapagliflozin, canagliflozin, empagliflozin, ipragliflozin, ertugliflozin, tofogliflozin, and luseogliflozin). Dapagliflozin recently received Food and Drug Administration approval for use in the treatment of heart failure in patients with or without type 2 diabetes (T2DM). Therefore, SGLT2 inhibitors have good future prospects in the field of biomedical research and development.

Phlorizin is the starting point for the development of specific SGLT2 inhibitors, driving research in this field (Osorio et al., 2010; Martus et al., 2022). Phloridzin is a naturally occurring non-selective SGLT2 inhibitor, which was first associated with diabetes in the 1980s, when found to correct hyperglycemia and normalize insulin sensitivity without altering insulin levels in diabetic rats (Rossetti et al., 1987). However, phlorizin is not an ideal therapeutic drug in diabetes treatment owing to its poor solubility in water, poor oral bioavailability, and non-selective SGLT2 inhibition (Choi, 2016). During subsequent decades, six subtypes of SGLT were identified in the human body. Among these, SGLT2 exhibits low affinity and high glucose transport capacity and is almost entirely located in the proximal tubule epithelium (Hummel et al., 2011). In healthy individuals, more than 90% of filtered glucose is reabsorbed by SGLT2 in the proximal tubule. However, SGLT2 inhibitors currently block less than 50% of renal glucose resorption in healthy individuals; the urine glucose levels are maintained by SGLT2 and do not increase even in patients with diabetes (List et al., 2009; Liu et al., 2012). Moreover, although SGLT2 inhibitors have additional benefits on cardiovascular and kidney systems, in addition to hypoglycemia through renal glucose elimination, the exact molecular mechanisms underlying these processes remain unclear. Thus, further in-depth research on SGLT2 inhibitors through clinical trials and animal experiments is crucial for updating current knowledge and developing inhibitors that are more effective inhibitors. Although these studies provide preliminary insight into the field of SGLT2 inhibitors, a more comprehensive bibliometric analysis of SGLT2 inhibitors is not yet available.

Recently, scholars have contributed to the authoritative guidance on bibliometric analysis, describing its value and multiple major contributions in advancing theory and practice, such as promoting objective discovery of knowledge clusters and promoting objective assessment and reporting of research productivity and impact (Lim et al., 2022; Mukherjee et al., 2022). Bibliometric analysis has been widely used in the quantitative analysis of academic literature to describe the hotspots, trends, and contributions of scholars, journals, countries, and regions (Donthu et al., 2021). Bibliometric studies tend to be more objective and extensive in scope than other types of reviews, and can reveal the evolutionary nuances of a specific field as well as the emerging areas in that field (Chen et al., 2014; Guler et al., 2016; Taj et al., 2019). Bibliometric analysis has also been more widely applied to biomedicine and healthcare (Zhao et al., 2018; Ahmadvand et al., 2019; Sugimoto et al., 2019; Xu et al., 2021; Ma et al., 2022). These bibliometric studies summarize current research hotspots and provide future research directions of a particular field. In recent years, researchers have made great progress in the study of SGLT2 inhibitors and published a large number of research results. However, to the best of our knowledge, few bibliometric analyses of the global research trends in SGLT2 inhibitors have been published. Therefore, we believe that it is necessary and urgent for bibliometrics to explore and discover the current research status and frontier trends of SGLT2 inhibitors. We hope that this study will provide valuable information to clinicians and researchers and provide new perspectives and a basis for future SGLT2 inhibitor research.

## 2 Data and methods

### 2.1 Data source and search strategy

**FIGURE 1:**
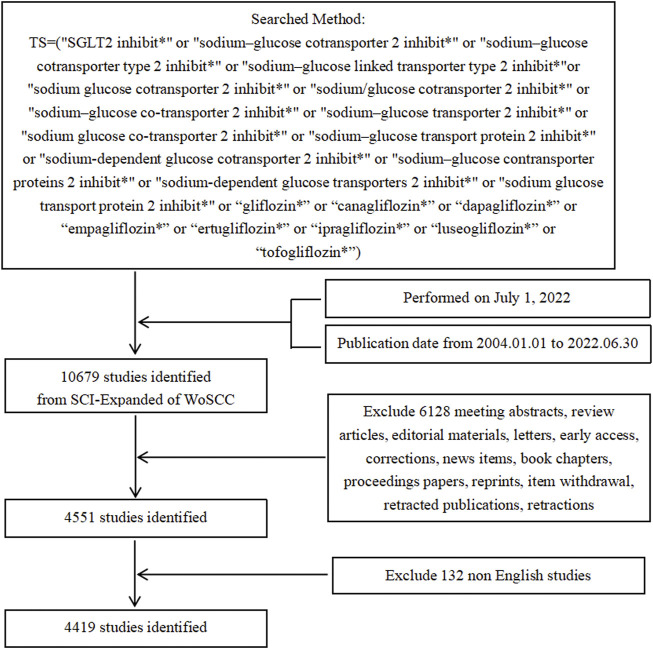
Flowchart of the screening process.

Data were downloaded from the Science Citation IndexExpanded (SCI-Expanded) database of the Web of Science Core Collection (WoSCC) on a single day, 1 July 2022. The search strategy was as follows: TS=(“SGLT2 inhibit*” or “sodium–glucose cotransporter 2 inhibit*” or “sodium–glucose cotransporter type 2 inhibit*” or “sodium–glucose linked transporter type 2 inhibit*”or “sodium glucose cotransporter 2 inhibit*” or “sodium/glucose cotransporter 2 inhibit*” or “sodium–glucose co-transporter 2 inhibit*” or “sodium–glucose transporter 2 inhibit*” or “sodium glucose co-transporter 2 inhibit*” or “sodium–glucose transport protein 2 inhibit*” or “sodium-dependent glucose cotransporter 2 inhibit*” or “sodium–glucose contransporter proteins 2 inhibit*” or “sodium-dependent glucose transporters 2 inhibit*” or “sodium glucose transport protein 2 inhibit*” or “gliflozin*” or “canagliflozin*” or “dapagliflozin*” or “empagliflozin*” or “ertugliflozin*” or “ipragliflozin*” or “luseogliflozin*” or “tofogliflozin*”). Only original articles written in English and published from 1 January 2004 to 30 June 2022 were included. A total of 4,419 records were obtained for further study. Detailed selective enrollment and screening procedures are illustrated as a flowchart in Figure 1.

### 2.2 Data collection and analysis

Bibliometric analysis typically involves the construction and graphical representation of bibliometric maps (Hou et al., 2018). The bibliometric structures are summarized using techniques for science mapping (e.g., citation analysis, co-citation analysis, co-occurrence analysis, and co-authorship analysis) and bibliometric analysis enhancement techniques (e.g., clustering, visualization) (Lim et al., 2022). Data including the title, year of publication, author names, nationality and affiliation, keywords, and abstract were downloaded from the SCI-Expanded of WoSCC in the form of TXT files and imported into Microsoft Excel 2019, CiteSpace (5.8.R3) and VOSviewer (1.6.18) for performing visual analysis. CiteSpace is a software for identifying and displaying new trends and developments in scientific literature (Chen, 2006). VOSviewer uses the visualization of similarities mapping technique (van Eck and Waltman, 2017) to show the most important terms from the acquired publications that belong to a cluster, as well as the co-occurrence between these terms (van Eck and Waltman, 2017).

In this study, the bibliometric analysis is mainly divided into three parts. First, VOSviewer was used to conduct co-authorship analysis of author, countries, regions and institutions, and create a visualization diagram to examine the scientific strength and international influence of these authors, countries, regions, and institutions in the field of SGLT2 inhibitor research. Second, VOSviewer was used to conduct co-citation analysis of reference and co-occurrence analysis of keywords, to create a visualization diagram. The document co-citation network reflects the academic influence of the literature and is used to establish the knowledge base of SGLT2 inhibitors. The keyword co-occurrence network visualization is a method for determining research hotspots and predicting research trends. Lastly, CiteSpace was used to detect keyword bursts, which added richer interpretations to the understanding of the emerging trends in the field of SGLT2 inhibitors.

## 3 Results

### 3.1 Global publication trends

**FIGURE 2:**
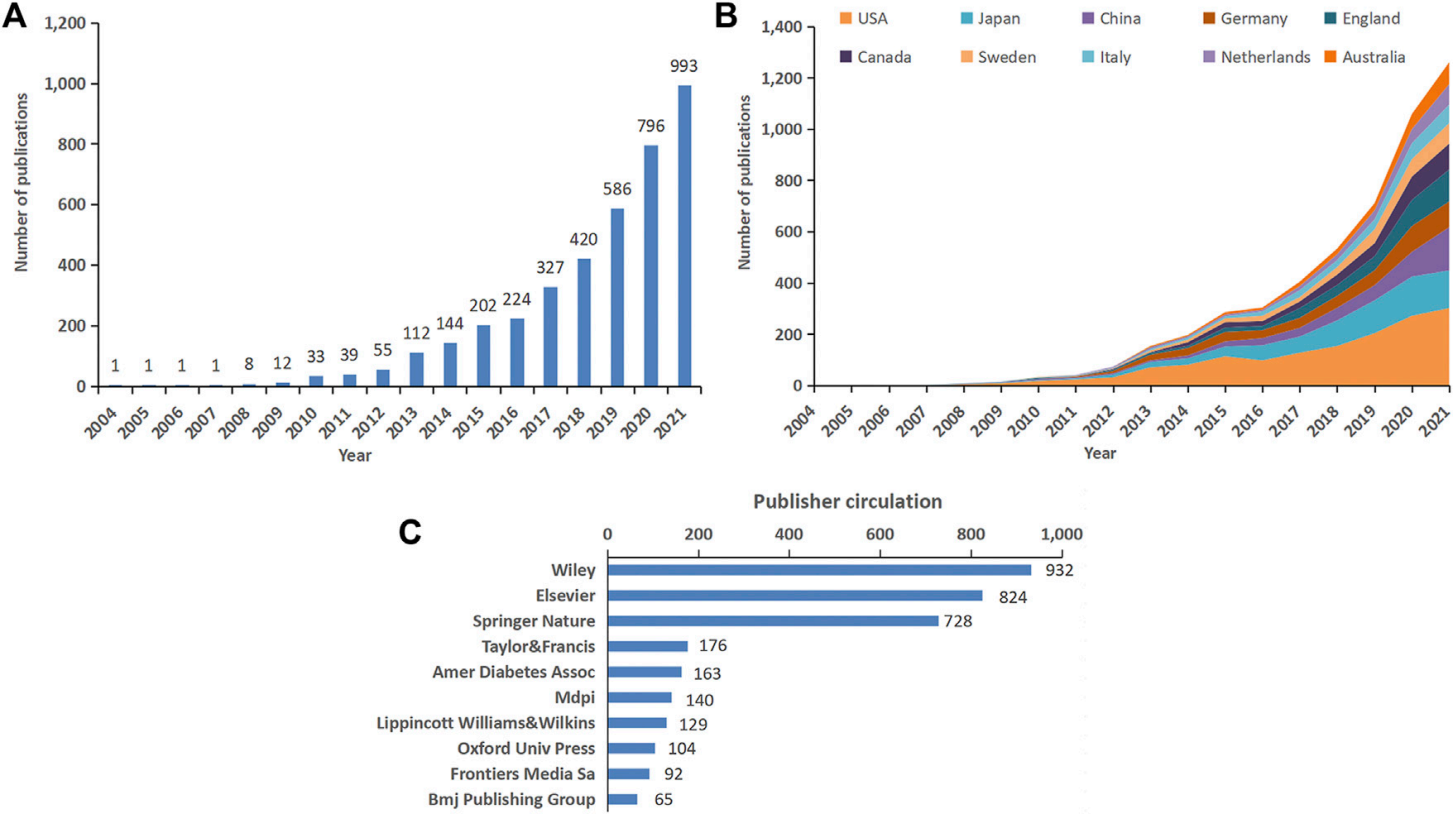
Global publication trend and country or region analysis for SGLT2 inhibitor research. **(A)** Annual worldwide publication output. **(B)** Growth trends in publication output from the top 10 productive countries. **(C)** Top 10 publishers according to their contribution to the total number of articles in SGLT2 inhibitor research.

Based on our search strategy, 4,419 original articles met the inclusion criteria. The annual publication output in the SGLT2 inhibitors field is shown in Figure 2A. The number of annual publications increased from 1 in 2004 to 993 in 2021. The number of annual publications began to exceed 200 in 2015 and then increased significantly each year, with a nearly five-fold increase in 2021. A total of 464 articles were published in the first half of 2022, but this does not reflect the total number of publications for the whole year. We further identified the annual national output of the 10 most productive countries and regions Figure 2B. The different colors show different countries, and the slope shows trends in the number of annual publications. United States ranked the first in the number of annual publications, followed by Japan and China. The top 10 publishers according to their contribution to the total number of articles on SGLT2 inhibitors are shown in Figure 2C. Wiley (932), Elsevier (824), and Springer Nature (728) far exceeded other publishers regarding the number of publications, confirming their prime international status in the publishing industry.

### 3.2 Distribution of source journals

The top 10 productive and co-cited journals on this topic are listed in Table 1. The top 10 journals published 1,181 publications, accounting for 26.73% of all publications, with most belonging to the category of endocrinology and metabolism. According to the 2021 Journal Citation Reports, half of the 10 most productive journals were classified in Q1, three were in Q2, and two were in Q3. All co-cited journals were located in Q1 regions. Diabetes Obesity and Metabolism published the most papers (406 articles, 9.19%), with the third highest number of co-citations (7,942), and an impact factor (IF) of 6.408 in 2021. Circulation published only 59 articles but had the highest IF (39.918) in 2021 and the fourth most co-citations (4,836). The most frequently co-cited journal was The New England Journal of Medicine (11,617), which had the second highest IF (176.079) in 2021. The Lancet had the highest IF (202.731), but ranked fifth among the most cited journals.

**TABLE 1:** Top 10 productive journals and co-cited journals in SGLT2 inhibitor research.

Rank	Productive journal	Count N/4,419 (%)	JCR	Citation	If 2021	Rank	Co-cited journal	JCR	Co-citation	If 2021
1	Diabetes Obesity and Metabolism	406 (9.19%)	Q1^a^	14,209	6.408	1	The New England Journal of Medicine	Q1^b^	11,617	176.079
2	Diabetes Care	131 (2.96%)	Q1^a^	14,448	17.152	2	Diabetes Care	Q1^a^	10,858	17.152
3	Cardiovascular Diabetology	128 (2.90%)	Q1^a^ ^,^ ^c^	4,666	8.949	3	Diabetes Obesity and Metabolism	Q1^a^	7,942	6.408
4	Diabetes Therapy	126 (2.85%)	Q3^a^	1,319	3.595	4	Circulation	Q1^c^ ^,^ ^d^	4,836	39.918
5	Journal of Diabetes Investigation	95 (2.15%)	Q3^a^	1,162	3.681	5	The Lancet	Q1^b^	3,493	202.731
6	Diabetes Research and Clinical Practice	73 (1.65%)	Q1^a^	778	8.180	6	Diabetologia	Q1^a^	3,450	10.460
7	Circulation	59 (1.34%)	Q1^c^ ^,^ ^d^	6,382	39.918	7	Diabetes	Q1^a^	3,154	9.337
8	Scientific Reports	59 (1.34%)	Q2^d^	1,137	4.996	8	Cardiovascular Diabetology	Q1^a^ ^,^ ^c^	2,594	8.949
9	Clinical Therapeutics	53 (1.20%)	Q2^c^ ^,^ ^d^	1,015	3.637	9	The Lancet Diabetes and Endocrinology	Q1^a^	2,552	44.867
10	PLoS One	51 (1.15%)	Q2^a^	1,829	3.752	10	Journal of the American College of Cardiology	Q1^c^	2,237	27.203

^a^ Endocrinology and Metabolism.

^b^ Medicine, General and Internal.

^c^ Cardiac & Cardiovascular systems.

^d^ Pharmacology and Pharmacy.

IF, impact factor; JCR, Journal Citation Reports.

### 3.3 Distribution characteristics of country/region and institution

Analysis of the county, region, or institution in the distribution of publications can provide abundant information for researchers about the countries, regions, or institutions at the frontiers of research. In our study, 4,419 articles came from 93 countries or regions and 5,518 institutions. We then identified the 10 most productive countries, regions and institutions involved in this research (Table 2). The United States had the largest number of articles (1,629, 36.86%), followed by Japan (817, 18.49%) and China (586, 13.26%). The United States had 88,892 citations, which ranked first among all included countries/regions. Netherlands had the most citations per article (117) but ranked ninth in the number of articles (274). The institution with the most publications was AstraZeneca (272), followed by the University of Toronto (244) and Boehringer Ingelheim Pharma GmbH & Co. KG (207).

**TABLE 2:** Top 10 countries/regions and institutions in SGLT2 inhibitor research.

Rank	Country/region	Count N/4,419 (%)	Citations per article	Total citations	Rank	Institution	Count N/4,419 (%)	Country/region	Citations per article	Citations
1	United States	1,629 (36.86%)	55	88,892	1	AstraZeneca	272 (6.16%)	England	54	14,660
2	Japan	817 (18.49%)	38	31,327	2	Univ Toronto	244 (5.52%)	Canada	115	28,145
3	China	586 (13.26%)	39	22,747	3	Boehringer Ingelheim Pharma GmbH & Co. KG	207 (4.68%)	Germany	109	22,625
4	Germany	514 (11.63%)	78	39,987	4	Harvard Med Sch	168 (3.80%)	United States	85	14,227
5	England	470 (10.64%)	89	41,812	5	Univ Groningen	167 (3.78%)	Netherlands	102	17,052
6	Canada	407 (9.21%)	83	33,751	6	Janssen Res & Dev LLC	150 (3.39%)	United States	67	10,032
7	Sweden	335 (7.58%)	61	20,368	7	Brigham & Women’s hospital	132 (2.99%)	United States	87	11,520
8	Italy	303 (6.86%)	85	25,628	8	Univ Glasgow	109 (2.47%)	Scotland	74	8,033
9	Netherlands	274 (6.20%)	117	31,987	9	Univ Stanford	94 (2.13%)	United States	106	9,944
10	Australia	266 (6.02%)	93	24,623	10	Boehringer Ingelheim Pharmaceuticals Inc	90 (2.04%)	United States	115	10,355

Univ, University; Med, Medical; Sch, School; Res, Research; Dev, Development; Inc, Incorporated.

**FIGURE 3:**
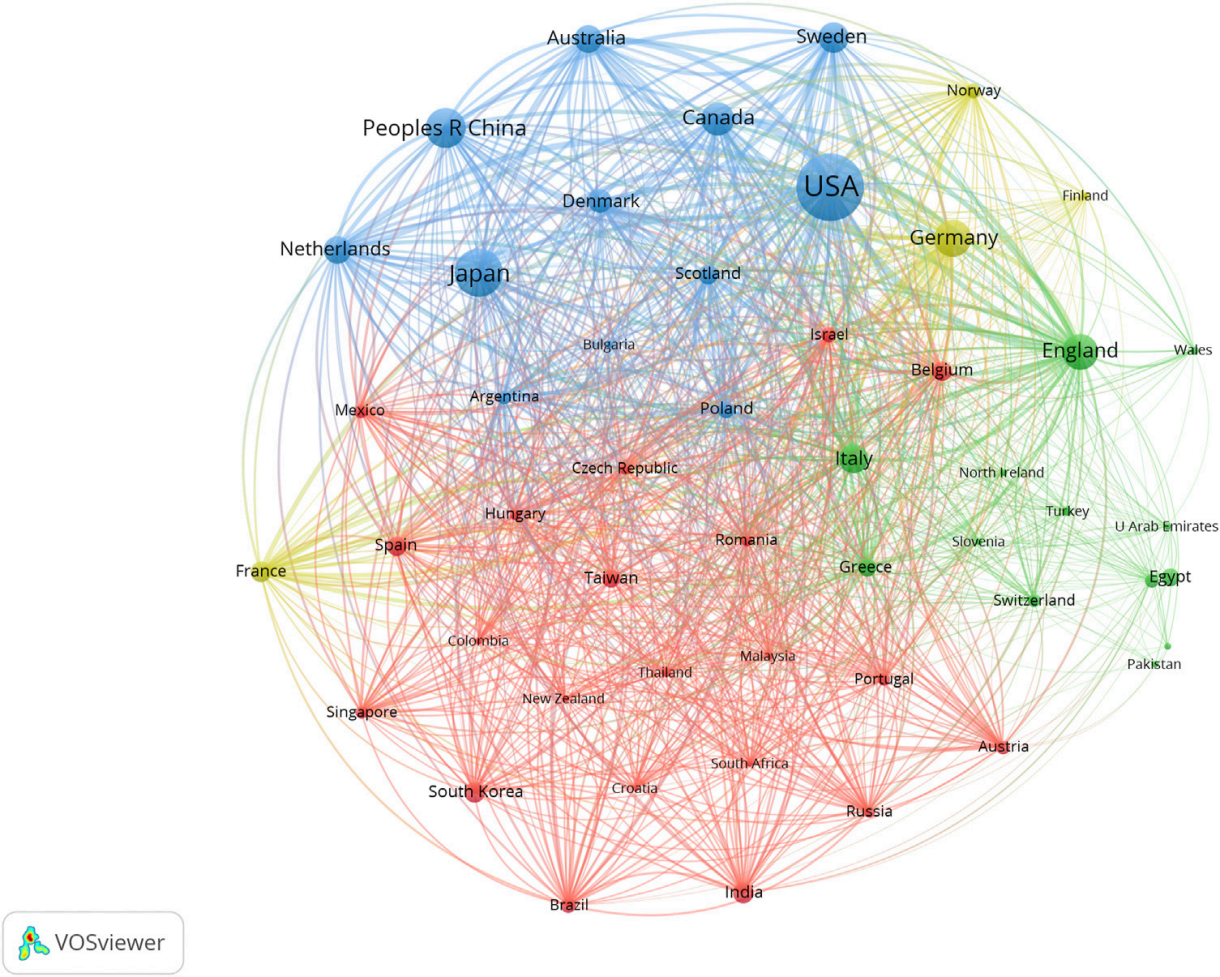
VOSviewer network visualization map of countries or regions involved in SGLT2 inhibitor research. Collaboration analysis of the top 50 of 93 countries or regions. Node size represents the co-authorship frequency, and the line between two nodes represents a collaboration between the two countries, regions, or institutions; the thicker the line, the closer the collaboration.

**FIGURE 4:**
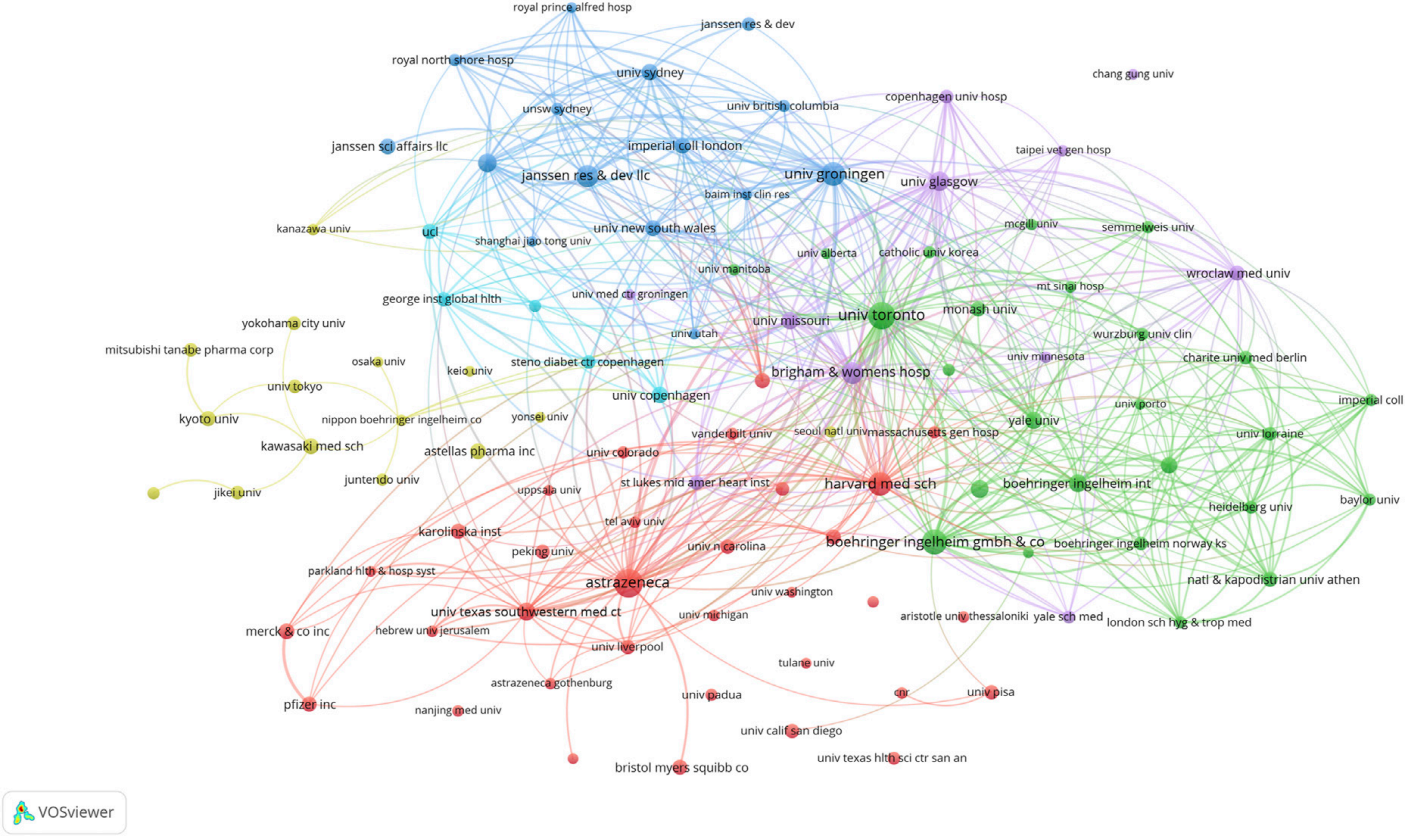
VOSviewer network visualization map of institutions involved in SGLT2 inhibitor research. Collaboration analysis of the top 105 of 5,518 institutions. Node size represents the co-authorship frequency, and the line between two nodes represents a collaboration between the two countries, regions, or institutions; the thicker the line, the closer the collaboration.

We used VOSviewer to construct a network visualization map of countries, regions and institutions involved in SGLT2 inhibitor research (Figures 3, 4). Countries, regions and institutions with similar colors represent clusters. Larger nodes represent countries, regions and institutions with higher productivity in this field. The links between nodes represent the cooperative relationship. Figure 3 shows the 50 most productive countries or regions. The blue cluster shows that the United States had the largest node and the highest total link strength, indicating that it participated in the most collaborations with other countries worldwide; for instance, the United States had close cooperation with Japan, China, and Canada. The green cluster was led by England collaborating with Italy, Egypt, and Greece. The yellow cluster was led by Germany collaborating with France, Norway, and Finland. The red cluster included South Korea, India, Spain, and Belgium. Figure 4 shows the collaboration network of the top 105 institutions. AstraZeneca, the University of Toronto, and Boehringer Ingelheim Pharma GmbH & Co. KG had the highest total link strength, revealing close collaboration with many other institutions.

### 3.4 Distribution and co-authorship of authors

**FIGURE 5:**
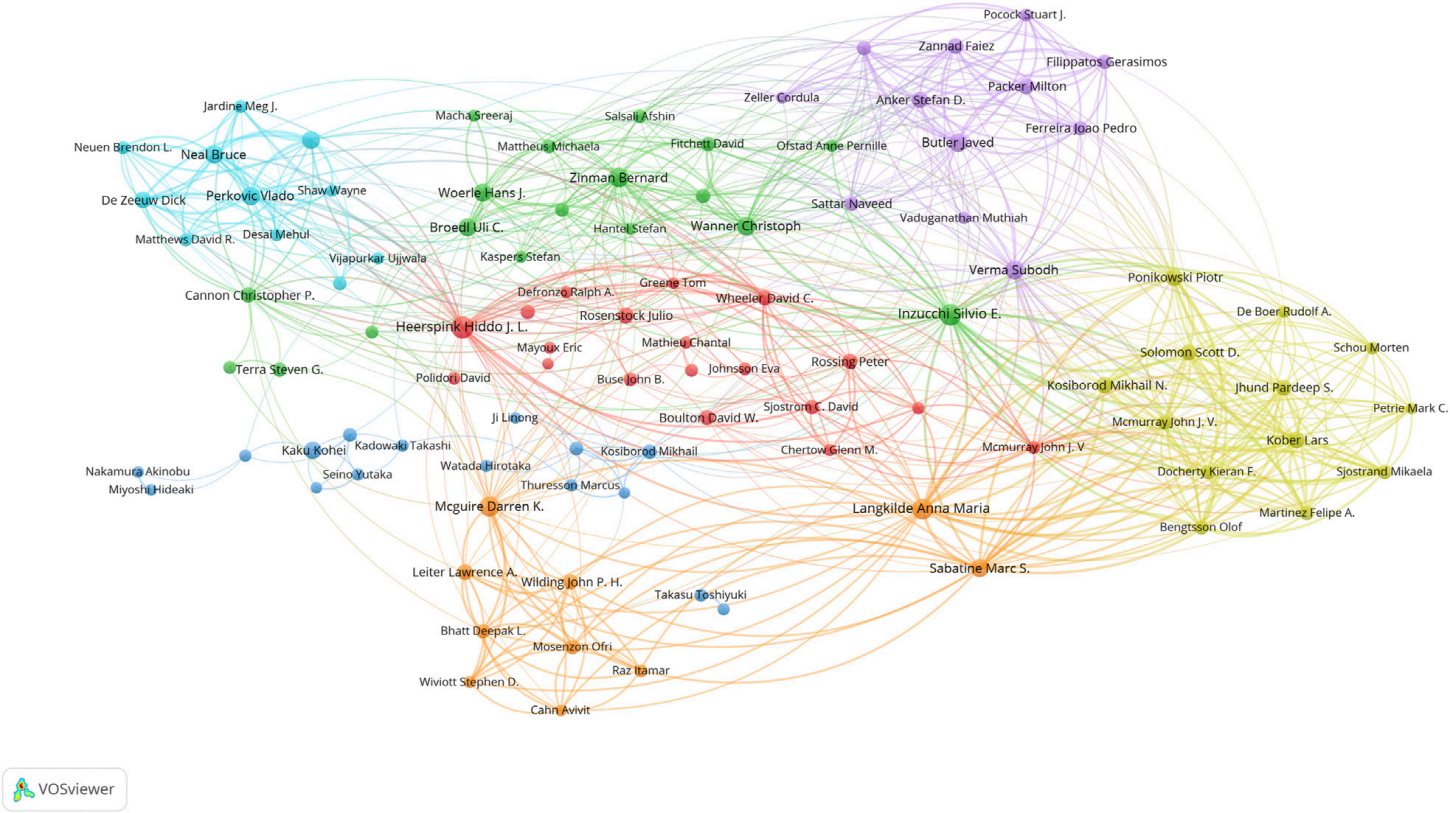
VOSviewer network visualization map of authors involved in SGLT2 inhibitor research. Collaboration analysis of the top 100 authors. Node size represents the co-authorship frequency, and the line between two nodes represents a collaboration between the two authors; the thicker the line, the closer the collaboration.

The 10 most productive authors and co-cited authors contributing to publications are shown in Table 3. Heerspink HJL published 98 articles, ranking first in the number of publications, followed by Inzucchi SE (89 articles) and Langkilde AM (77). The most frequently co-cited author contributing to publications was Zinman B (1,784 citations), followed by Neal B (1,477), Ferrannini E (1,265), and Heerspink HJL (1,128). The collaboration visualization network of authors formed by VOSviewer is shown in Figure 5. In total, 100 authors have published at least 20 publications. Each node represents an author, where the size of the node represents the number of publications. Each link indicates collaboration, where the thickness of the link between nodes shows the relative strength of the relationship. The most significant co-author networks involved Heerspink HJL, Inzucchi SE, Langkilde AM, McGuire DK, and Zinman B.

**TABLE 3:** Top 10 productive authors and co-cited authors in SGLT2 inhibitor research.

Rank	Author	Article counts	Citation per article	Rank	Co-cited author	Co-citations
1	Heerspink HJL	98	48	1	Zinman B	1,784
2	Inzucchi SE	89	149	2	Neal B	1,477
3	Langkilde AM	77	49	3	Ferrannini E	1,265
4	McGuire DK	73	83	4	Heerspink HJL	1,128
5	Zinman B	67	166	5	Rosenstock J	1,119
6	Wanner C	64	164	6	Wiviott SD	921
7	Perkovic V	61	115	7	McMurray JJV	874
8	Verma S	61	59	8	Packer M	800
9	Butler J	59	60	9	Inzucchi SE	775
10	Broedl UC	58	239	10	DeFronzo RA	751

### 3.5 Analysis of co-cited references

**FIGURE 6:**
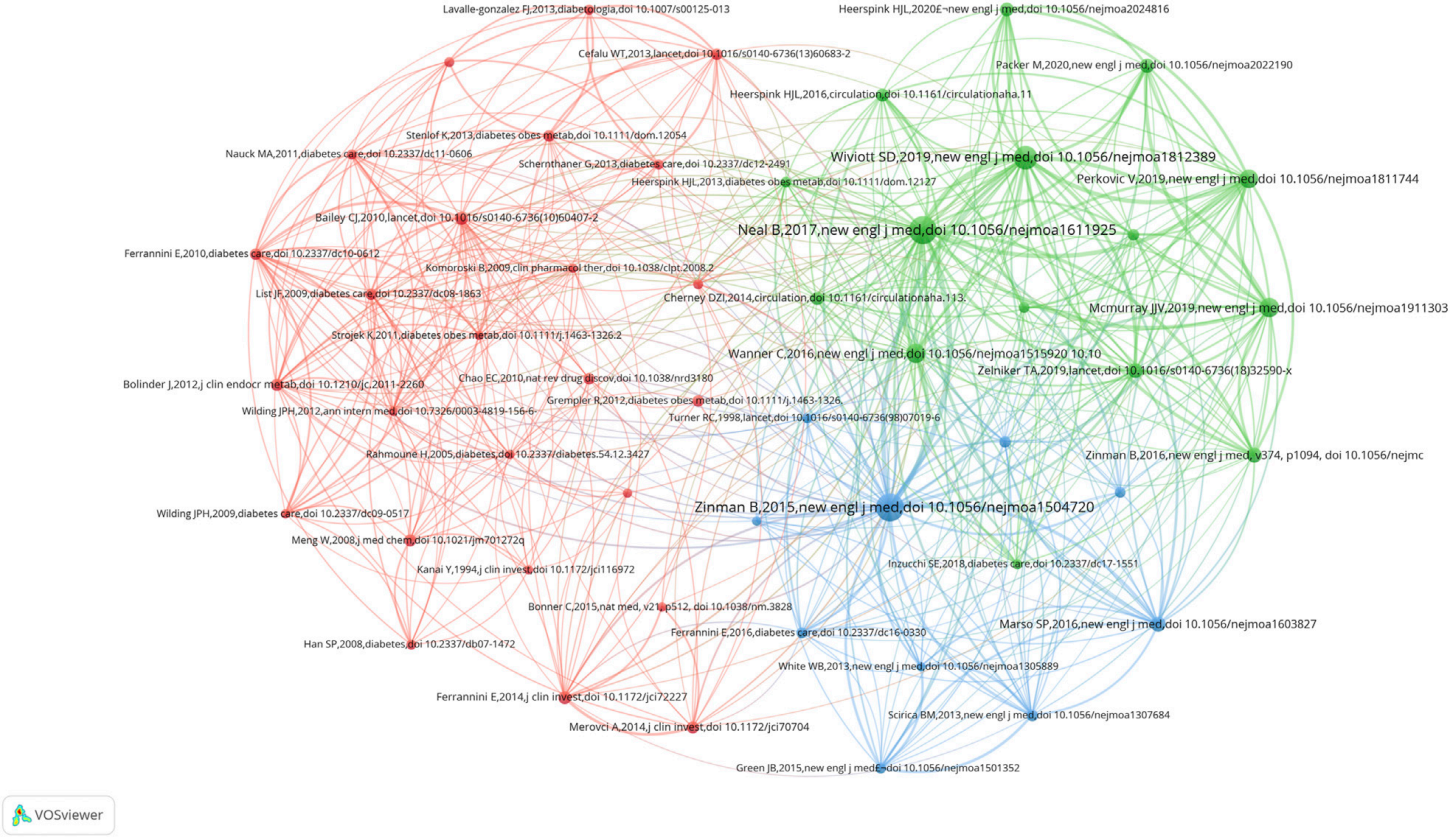
VOSviewer network visualization map of the top 50 cited references. Each node represents a reference, where the size of the node represents the citation counts, and each link between references indicates that the two references were co-cited, with a thicker line indicating a closer relationship.

Co-cited references represent the frequency of two publications being cited together by other publications. The network map of co-cited references consisted of the top 50 references as shown in Figure 6. In general, the more citations, the greater is the co-cited frequency. Table 4 shows the top 10 articles that have been highly cited in SGLT2 inhibitor studies. Nine of the top 10 cited references were published in authoritative journals with high impact factors, such as The New England Journal of Medicine. Literatures hotspots included mainly on clinical trials of SGLT2 inhibitors on cardiovascular and renal outcomes. The most co-cited paper was “Empagliflozin, Cardiovascular Outcomes, and Mortality in Type 2 Diabetes,” published by Zinman B et al. (2015), with 1,236 citations. The next most co-cited paper was “Canagliflozin and Cardiovascular and Renal Events in Type 2 Diabetes,” published by Neal B et al. (2017) (1,216 citations).

**TABLE 4:** Top 10 co-cited references in SGLT2 inhibitor research.

Rank	Co-citations	Author	Title	Year	Journal	DOI	RCTs
1	1,236	Zinman B	Empagliflozin, cardiovascular outcomes, and mortality in type two diabetes	2015	NEJM	10.1056/NEJMoa1504720	EMPA-REG OUTCOME ClinicalTrials
2	1,216	Neal B	Canagliflozin and cardiovascular and renal events in type two diabetes	2017	NEJM	10.1056/NEJMoa1611925	CANVAS and CANVAS-R ClinicalTrials
3	856	Wiviott SD	Dapagliflozin and cardiovascular outcomes in type two diabetes	2019	NEJM	10.1056/NEJMoa1812389	DECLARE–TIMI 58 ClinicalTrials
4	572	Wanner C	Empagliflozin and progression of kidney disease in type two diabetes	2016	NEJM	10.1056/NEJMoa1515920	EMPA-REG OUTCOME ClinicalTrials
5	571	McMurray JJV	Dapagliflozin in patients with heart failure and reduced ejection fraction	2019	NEJM	10.1056/NEJMoa1911303	DAPA-HF ClinicalTrials
6	561	Perkovic V	Canagliflozin and renal outcomes in type two diabetes and nephropathy	2019	NEJM	10.1056/NEJMoa1811744	CREDENCE ClinicalTrials
7	374	Zinman B	Empagliflozin, cardiovascular outcomes, and mortality in type two diabetes REPLY	2016	NEJM	10.1056/nejmc1600827	—
8	365	Zelniker TA	SGLT2 Inhibitors for primary and secondary prevention of cardiovascular and renal outcomes in type two diabetes: A systematic review and meta-analysis of cardiovascular outcome trials	2019	LANCET	10.1016/S0140-6736 (18)32590-X	—
9	359	Marso SP	Liraglutide and cardiovascular outcomes in type two diabetes	2016	NEJM	10.1056/NEJMoa1603827	LEADER ClinicalTrials
10	336	Packer M	Cardiovascular and renal outcomes with empagliflozin in heart failure	2020	NEJM	10.1056/NEJMoa2022190	EMPEROR-Reduced ClinicalTrials

RCTs, randomized controlled trials; NEJM, New England Journal of Medicine.

### 3.6 Analysis of keyword co-occurrence clusters

**FIGURE 7:**
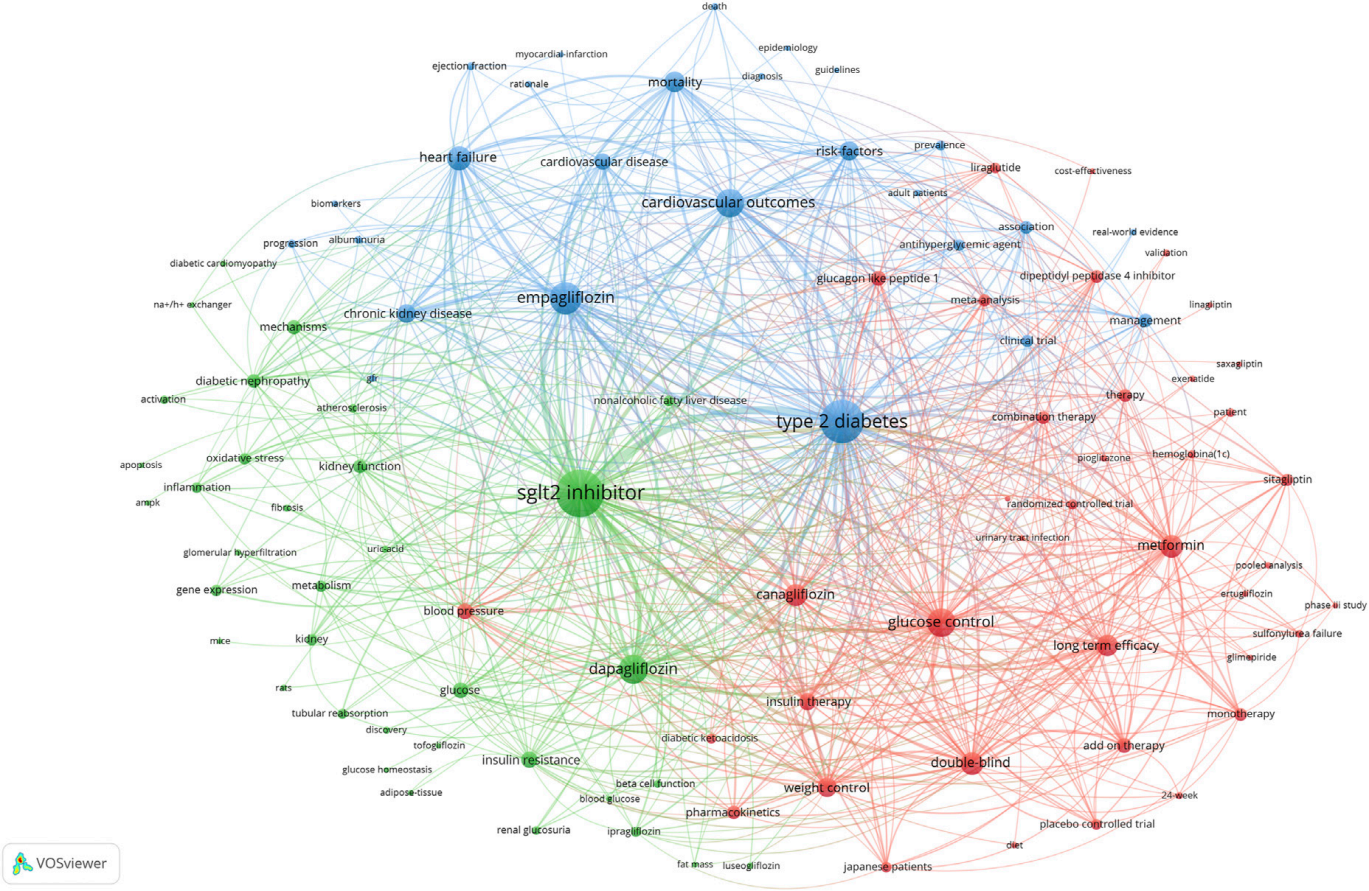
Co-occurrence network visualization map of the top 100 keywords. The visualization map of publications for 100 keywords forming three collaborating clusters (nodes with the same color); a node represents a keyword, the size of the node represents the number of publications, a link shows collaboration, and the distance and the thickness of the link between nodes show the relative strength of the relation.

**FIGURE 8:**
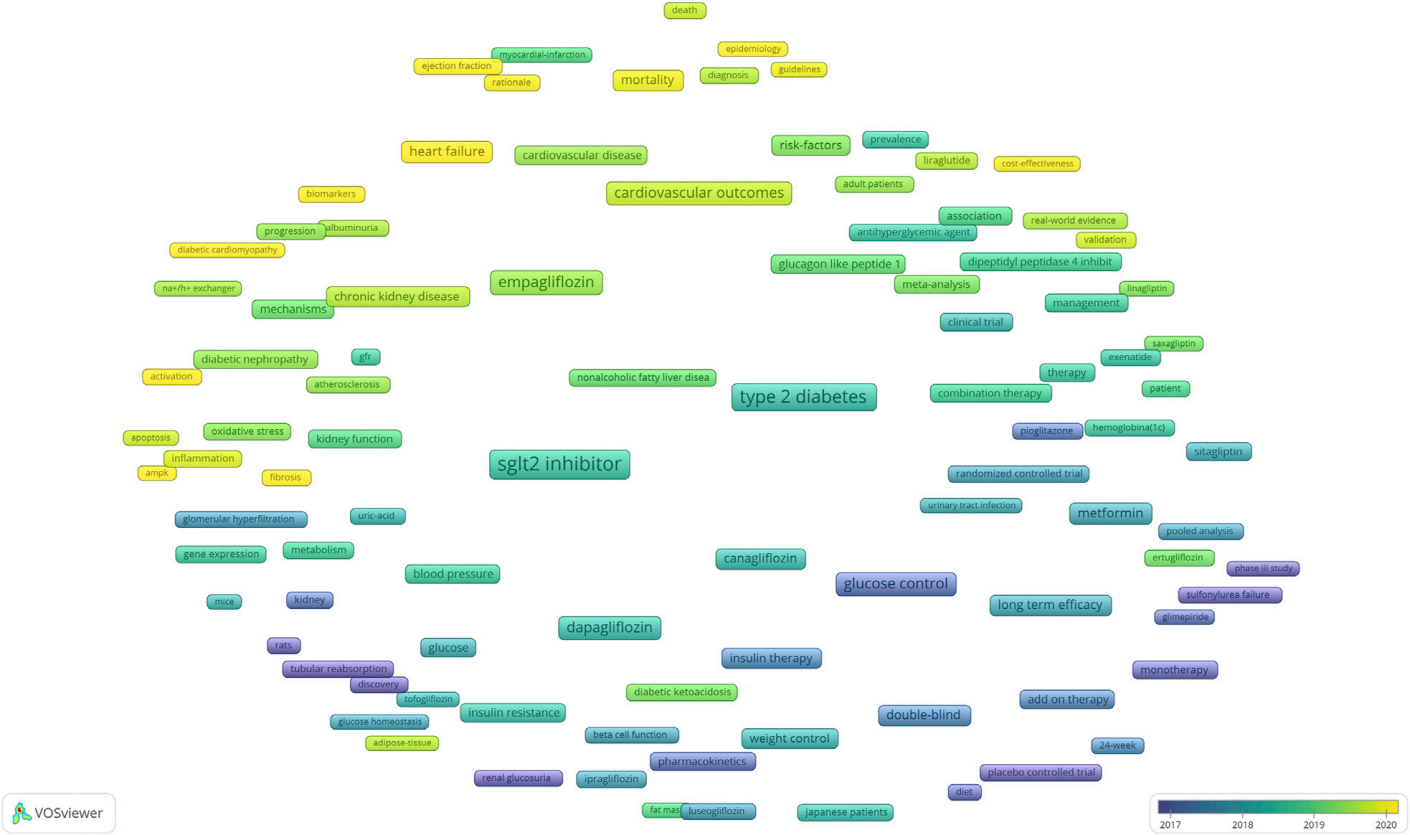
Co-occurrence overlay visualization map of the top 100 keywords. The color of the node represents the average publication year of the keywords, and the blue to yellow represents the average publication year from early to late.

When two keywords appear in the same publication, a co-occurrence relationship is formed between them. We used VOSviewer to construct a co-occurrence network visualization map of the top 100 keywords (Figure 7). There were three clusters: SGLT2 inhibitor therapy (red), clinical trials (blue), and related mechanisms (green). The more frequently the keyword appears, the larger the label and circle of the keyword. In overlay visualization, keywords were colored differently according to their average publication year (Figure 8). The color of the node represents the average publication year of the keywords, and the color scale from blue to yellow represents the average publication year from early to late. For instance, “glucose control” and “therapy” peared at the beginning of the discovery of this area, whereas the keywords “heart failure,” “diabetic cardiomyopathy” “ejection fraction,” “mortality,” “biomarker,” “fibrosis,” “ampk,” and “guideline” were more recent.

### 3.7 Analysis of burst keywords

**FIGURE 9:**
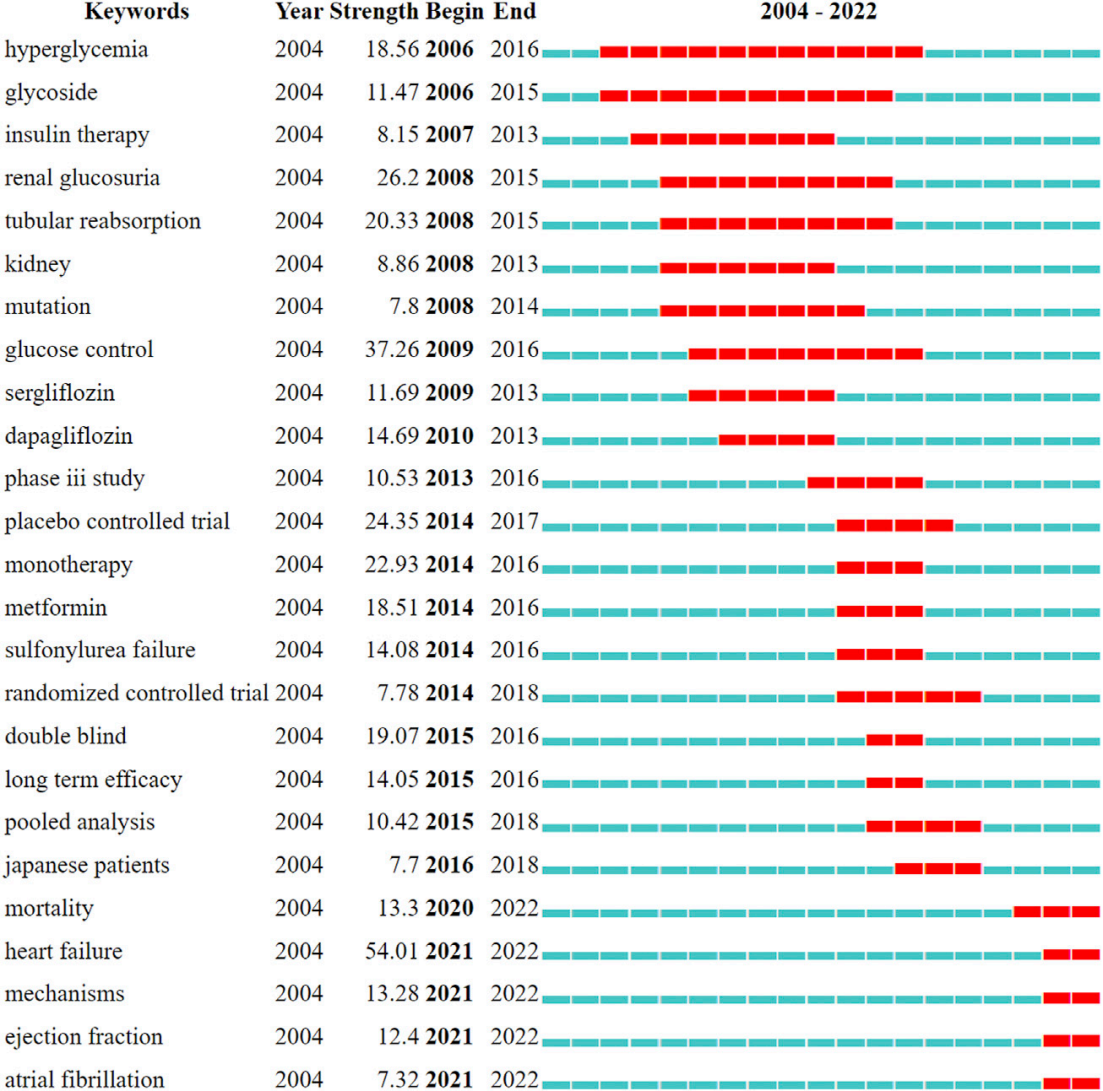
Top 25 keywords with citation burst (sorted by the beginning year of the burst). The years between “Begin” and “End” represent the period when the keyword was more influential. The dark blue bar represents the year in which the keyword appeared, and the red bar refers to the burst duration. Burst strength indicates the importance of keywords to the research field.

CiteSpace was further used to detect burst keywords with high frequency and therefore determine research hotspots and frontiers over time. The top 25 keywords with the strongest citation bursts (sorted by the beginning year of the burst) are shown in Figure 9. Keywords such as “mortality” (strength 13.3), “heart failure” (strength 54.01), “mechanism” (strength 13.28), “ejection fraction” (strength 12.4), and “atrial fibrillation” (strength 7.32), were the most recent trending keywords.

## 4 Discussion

Bibliometric and visual analysis can present the current research status and predict promising research fields. In this study, we performed a bibliometric analysis of SGLT2 inhibitor-related studies from 2004 to the first half of 2022 using SCI-Expanded of WoSCC to fully understand global research trends and hotspots and provide references for researchers in this field.

### 4.1 Global trends in SGLT2 inhibitor research

The trend of the annual number of publications is an important indicator of the development in an academic field. Our study revealed that a total of 4,419 original articles met the inclusion criteria, and articles published after 2015 accounted for 90% of all publications. Rapid growth in annual publications may be related to the completion of several high-quality multicenter randomized controlled trials (RCTs) in recent years, such as the EMPA-REG OUTCOME clinical trial published by Zinman B in 2015, which was the most cited, followed by the CANVAS project (CANVAS and CANVAS-r Clinical Trials) published by Neal B in 2017 (Zinman et al., 2015; Neal et al., 2017). These two trials are the most basic and important research, which have greatly promoted the development in the field. The DECLARE-TIMI 58 clinical trial, DAPA-HF trial and CREDENCE trial in 2019 also strongly increased the annual publication output (McMurray et al., 2019; Perkovic et al., 2019; Wiviott et al., 2019). As a result, rapid growth in publication output is expected to continue.

Among the 10 most active journals in SGLT2 inhibitor research, four were also among the top 10 co-cited journals (Diabetes Obesity and Metabolism, Diabetes Care, Cardiovascular Diabetology, and Circulation). Among these, the IF values of Diabetes Care and Circulation were greater than 10, indicating outstanding contributions in the field of SGLT2 inhibitor research. The most frequently co-cited journals were The New England Journal of Medicine and Diabetes Care. Notably, nine of the top 10 co-cited references were published in The New England Journal of Medicine, and the main research hotspots were the effect of SGLT2 inhibitors on clinical trials of cardiovascular and kidney outcomes, reflecting the extensive and professional coverage of authoritative articles.

### 4.2 Co-authorship networks in SGLT2 inhibitor research

The degree of collaboration between countries/regions, institutions, and authors was assessed by co-authorship analysis. Collaborative research networks can highlight potential opportunities for strengthening collaboration within and outside existing networks and provide potential collaborative opportunities for other groups. Currently, the United States ranks the first in terms of the number of publications, total IF, and total number of citations in the field of SGLT2 inhibitor research, with the total number of citations far greater than that in other countries or regions. Undoubtedly, the United States makes the greatest contribution and can be considered a leader in this field. Despite the relatively low total publication outputs of other countries or regions such as Japan, China, Germany, England, and Canada, their rapid growth in annual output in recent years reflects tremendous progress in this area. Currently, these countries, led by the United States, are in a leading position in SGLT2 inhibitor research, thereby promoting development in this field. Notably, China ranked third for the total number of publications but ranked ninth for the total citations and citations per article, indicating that the quality of research in the field of SGLT2 inhibitors should be improved. Interestingly, Netherlands had the second-lowest total number of publications among the top 10 countries but was the most cited country per article, suggesting that the quality of research on SGLT2 inhibitors in Netherlands is very high; this could have an important impact on future development in this field. The discrepancy between the quantity and quality of studies may be attributed to the lack of a standardized academic evaluation system and high-quality multicenter RCTs, as well as uneven research capacities.

The top 10 institutions are at the heart of almost every collaborative network. Five of the top 10 institutions for SGLT2 inhibitor research are based in United States, thereby maximizing regional advantages and demonstrating the dominance of United States in the field. This may partly explain why United States consistently maintains a high quantity and quality of publications. AstraZeneca in England was the most productive institution worldwide, followed by the University of Toronto in Canada, indicating that these two institutions participated in the most collaborations with other institutions worldwide. Although Japan and China ranked second and third in terms of total publications, none of their research institutions ranked in the top 10, indicating a lack of institutions with professional and research stature in SGLT2 inhibitor research. The most effective organizations and groups are leading the trends in SGLT2 inhibitor research; thus, further study at these institutions will ensure continuous future development in this field.

Regarding the authors, we observed abundant exchange among authors, forming a large network of co-authors. Heerspink HJL produced the largest number of papers, whereas Zinman B had the most co-citations. These data and indicators provide us with the most academically influential and authoritative authors in the SGLT2 inhibitor field. The top 10 authors with the most publications were active researchers in this field. Three of the top 10 most prolific authors (Heerspink HJL, Inzucchi SE, and Zinman B) were among the top 10 most co-cited authors, suggesting that they considered not only the quantity but also the quality of their articles. Heerspink HJL focused on the cardiovascular and renal effects, potential mechanisms, and clinical applications of SGLT2 inhibitors in the treatment of diabetes mellitus, especially in the kidney (Heerspink et al., 2016; Heerspink et al., 2017; Heerspink et al., 2019). Furthermore, this author has studied the effect of dapagliflozin in patients with chronic kidney disease, with or without T2DM in 2020, through the DAPA-CKD clinical trial (Heerspink et al., 2020). To recognize pivotal development and frontier trends, articles by those authors are attributed greater reference value. Promoting collaboration among authors, institutions, and countries will increase the number of regularly published authors in this field and more effectively improve the global popularity and scope of SGLT2 inhibitor research.

### 4.3 Basic knowledge and hotspots in SGLT2 inhibitor research

#### 4.3.1 Analysis of co-cited references

Published articles that are cited frequently possess tremendous academic influence. In our study, the top co-cited references were used to investigate the knowledge base for SGLT2 inhibitors. Among the top 10 co-cited references, eight were RCTs, including seven clinical studies of SGLT2 inhibitors but only one clinical study of glucagon-like peptide one receptor agonists. In recent years, the research on SGLT2 inhibitors has focused on clinical studies of cardiovascular and renal outcomes. Specifically, SGLT2 inhibitors such as empagliflozin, canagliflozin, and dapagliflozin were the most widely studied hypoglycemic drugs in clinical trials (Zinman et al., 2015; Fitchett et al., 2016; Neal et al., 2017; Wiviott et al., 2019).

The EMPA-REG OUTCOME trial was recognized as a critical milestone, with the highest number of co-citations. The results of this trial indicated a reduction in the relative risk of death from cardiovascular causes (38%), death from any cause (32%), hospitalization for heart failure (35%), incident or worsening nephropathy (39%), renal-replacement therapy (55%), and doubling of the serum creatinine level (44%) among patients with T2DM at high risk of cardiovascular events who received empagliflozin (Zinman et al., 2015; Fitchett et al., 2016; Wanner et al., 2016). The EMPEROR-Reduced trial further confirmed that patients with heart failure (with or without diabetes) in the empagliflozin group had a lower risk of cardiovascular death or hospitalization for heart failure than patients in the placebo group (Packer et al., 2020). The CANVAS program (CANVAS and CANVAS-R clinical trials) showed that patients with T2DM and an elevated risk of cardiovascular disease who were treated with canagliflozin had a lower risk of major adverse cardiovascular events (MACE), including a composite of death from cardiovascular causes, non-fatal myocardial infarction, or non-fatal stroke, than patients treated with a placebo (Neal et al., 2017). The results also showed reductions in the relative risk of hospitalization for heart failure (33%) and progression of albuminuria (27%) and showed the composite outcome of a sustained 40% reduction in the estimated glomerular filtration rate, renal-replacement therapy, or renal death (Neal et al., 2017; Perkovic et al., 2018). Results from the DECLARE-TIMI 58 trial, which had the highest number of patients with multiple risk factors, showed that dapagliflozin resulted in a lower rate of cardiovascular death or hospitalization for heart failure but did not result in a lower or higher rate of MACE than the placebo among patients with T2DM who had, or were at risk for, atherosclerotic cardiovascular disease (Wiviott et al., 2019). Notably, the first large phase-3 trial of SGLT2 inhibitors enrolling patients without diabetes was the DAPA-HF trial, which showed that, in patients with heart failure and reduced ejection fraction, those receiving dapagliflozin had a lower risk of worsening heart failure or death from cardiovascular causes, regardless of the presence of diabetes (McMurray et al., 2019). Additionally, compared to completed SGLT2 inhibitor cardiovascular outcome trials (Zinman et al., 2015; Neal et al., 2017; Wiviott et al., 2019), the CREDENCE trial enrolled people at high risk for kidney failure and had a primary outcome of major renal end points and demonstrated that patients with T2DM and kidney disease who received canagliflozin had a lower risk of kidney failure and cardiovascular events than those who received a placebo at a median follow-up of 2.62 years (Perkovic et al., 2019). A series of clinical trials on the cardiovascular outcomes of SGLT2 inhibitors showed a significant reduction in hospitalizations for heart failure, regardless of the patients’ history of atherosclerotic cardiovascular disease or heart failure. Similarly, the progression of kidney disease was also evident in patients with and without atherosclerotic cardiovascular disease. A meta-analysis of SGLT2 inhibitors had the largest and most consistent effect in reducing the relative risk of heart failure hospitalization (31%) and kidney disease progression (45%), however, SGLT2 inhibitors reduced MACE by 11%, with the benefits observed only in patients with atherosclerotic cardiovascular disease (Zelniker et al., 2019).

#### 4.3.2 Keyword analysis

Keywords with strong co-occurrence relationships can more accurately reveal the main research interests and hotspots in the field than a single keyword, thereby helping researchers to detect research directions from numerous studies. By analyzing the results of burst keywords such as “heart failure,” “ejection fraction,” “mortality,” and “mechanism,” as well as the keyword co-occurrence clusters terms such as clinical trials, mechanisms, and therapy, we revealed that recent clinical trials of SGLT2 inhibitors have mainly focused on cardiovascular outcomes and mortality, especially in heart failure. The cardioprotective effects of SGLT2 inhibitors have been supported by several large placebo-controlled trials, with or without diabetes. For example, the EMPA-REG OUTCOME, CANVAS program, and DECLARE-TIMI 58 trials have all showed reductions in mortality and heart failure hospitalization patients with T2DM after treatment with SGLT2 inhibitors (Fitchett et al., 2016; Neal et al., 2017; Fitchett et al., 2019; Wiviott et al., 2019). The EMPEROR-Reduced and DAPA-HF trials showed that SGLT2 inhibitors treatment reduced the mortality and heart failure hospitalizations in patients with heart failure and a reduced ejection fraction, regardless of the diagnosis of diabetes (McMurray et al., 2019; Packer et al., 2020). SGLT2 inhibitors are, thus, promising new treatment options. Biomarkers predicting treatment response, either alone or in combination could optimize the success of SGLT2 inhibitor therapy (Akerblom et al., 2019; Nobbenhuis et al., 2021; Vaduganathan et al., 2022). However, the mechanism of these positive effects of SGLT2 inhibitors remains unclear.

Further combined with the overlay visualization analysis of co-occurring keywords such as “heart failure,” “diabetic cardiomyopathy” “ejection fraction,” “fibrosis,” and “ampk,” the potential molecular mechanism of SGLT2 inhibitors in heart failure and diabetic cardiomyopathy has become a research hotspot in recent years. The characteristic changes in the early stage of diabetic cardiomyopathy are myocardial and interstitial fibrosis, leading to diastolic and subsequent systolic dysfunction, and eventually develops into clinical heart failure (Jia et al., 2018). Currently, SGLT2 inhibitors mainly have the following potential mechanisms for cardio protection. 1) SGLT2 inhibitors can improve cardiac diastolic ability. A study showed that SGLT2 inhibitors improved Ca^2+^ homeostasis by increasing phospholamban phosphorylation and enhancing sarcoplasmic reticulum calcium ATPase 2a function, thereby further improving cardiac diastolic function in diabetic mice (Hammoudi et al., 2017). Another study showed that SGLT2 inhibitors significantly reduced diastolic tension in mouse myocardium, with no change in systolic force, regardless of diabetes, suggesting independent effects from diabetic conditions (Pabel et al., 2018). 2) SGLT2 inhibitors can attenuate myocardial fibrosis. For example, SGLT2 inhibitors can protect against diabetic cardiomyopathy and myocardial fibrosis through suppressing fibroblast activation and endothelial to mesenchymal transition *via* AMPKα-mediated inhibition of transforming growth factor β/Smad signaling (Li et al., 2019; Tian et al., 2021). SGLT2 inhibitors also attenuate cardiac fibrosis *via* regulation of the macrophage phenotype by a reactive oxygen and nitrogen species/STAT3-dependent pathway (Lee et al., 2017). Furthermore, SGLT2 inhibitors attenuate fibrosis by significantly reducing the expression level of collagen 1 (Arow et al., 2020). 3) SGLT2 inhibitors can reduce myocardial inflammation and oxidative stress. Studies have shown that the AMPK pathway is the most important and closely related to SGLT2 inhibitors in diabetic cardiomyopathy, and AMPK activation may be a key point in inhibition of the anti-inflammatory effects of SGLT2 inhibitors (Ye et al., 2017; Uthman et al., 2018; Aragon-Herrera et al., 2019; Chen et al., 2020). SGLT2 inhibitors can effectively reduce the expression of reactive oxygen species in mitochondria and cytoplasm. In heart failure with preserved ejection fraction, elevated levels of nitric oxide synthase can reduce the occurrence of myocardial inflammation (Kolijn et al., 2021). Moreover, SGLT2 inhibitors attenuate myocardial oxidative stress *via* activation of the Nrf2/ARE signaling pathway, and protect cardiomyocytes from hyperglycemia-induced damage by inhibiting NADPH oxidase-mediated oxidative stress (Li et al., 2019; Xing et al., 2021). Future studies on SGLT2 inhibitors will likely continue to focus on heart failure and diabetic cardiomyopathy, and conduct more in-depth analysis of molecular mechanisms, especially those related to fibrosis and AMPK.

Treatment for T2DM includes Western therapies and traditional Chinese medicine (TCM). TCM treatments, including Chinese herbal medicine and acupuncture, have been applied in clinical settings to treat T2DM (Dou et al., 2021). In addition, Chinese patent medicine as adjuvant therapy can reduce the occurrence of MACE in patients with coronary heart disease angina pectoris, especially in men and middle-aged people (Liu et al., 2022). However, this conclusion needs further verification by prospective cohort studies in the future. TCM emphasizes improving symptoms and preventing secondary complications (Zheng et al., 2020; Chen et al., 2022; Zuo et al., 2022). Unlike TCM, Western therapies focus on blood glucose regulation and cardiovascular risk factor management in patients with T2DM. Among them, SGLT2 inhibitors are the latest pharmacological development in the history of T2DM treatment. The keyword “guideline” is also a hot topic in recent years. In guideline-recommended drug therapy management, SGLT2 inhibitors with proven benefits are recommended for patients with chronic kidney disease or clinical heart failure and atherosclerotic cardiovascular disease (Davies et al., 2018). SGLT2 are no longer just for glycemic control, but a “booster” that transforms the treatment and management of diabetes with cardiovascular and renal disease. In addition to the underlying mechanism of cardiac protection, substantial evidence has suggested that SGLT2 inhibitors also help to reverse the molecular processes related to inflammation, extracellular matrix turnover, and fibrosis in patients with diabetic nephropathy, with long-term protection of renal function (Gallo et al., 2016; Aroor et al., 2018; Zhang et al., 2018; Heerspink et al., 2019). The CREDENCE trial showed that canagliflozin was associated with a reduced incidence of serious and non-serious kidney-related adverse events in patients with T2DM and chronic kidney disease (Heerspink et al., 2022). Studies have also explored the heterogeneity of the effects of SGLT2 inhibition on cardiovascular and all-cause mortality in the EMPA-REG OUTCOME, CANVAS Program, DECLARE-TIMI 58, and CREDENCE trials. However, statistical evidence for the heterogeneity of the effect of SGLT2 inhibition on death outcomes across trials was not clearly explained, and differences in outcomes may be at least partly down to chance (Yu et al., 2021). Thus, evidence from these large-scale clinical studies show that SGLT2 inhibitors, as a new type of hypoglycemic drug, can significantly improve the cardiorenal outcomes of patients with T2DM in addition to hypoglycemia, achieving comprehensive benefits for the “sugar–heart–kidney” system.

### 4.4 Strengths and limitations

In this study, bibliometric methods were used to objectively and quantitatively analyze the existing literature on SGLT2 inhibitors. The findings and suggestions may help researchers and clinicians understand the global research trends in SGLT2 inhibitors. Nevertheless, there are some limitations to our study. Firstly, owing to the nature of the CiteSpace software, our data are filtered only from the SCI-Expanded of WoSCC database, which may not be sufficiently comprehensive and may lead to data omission. Secondly, some recently published articles might be discounted because of low citation frequency during the short period following publication. Finally, non-English articles were excluded from the database and analysis, potentially leading to a source bias.

## 5 Conclusion

Global research into SGLT2 inhibitors increased rapidly in recent years. Our results reveal research hotspots, key research directions, productive authors, countries, regions and institutions focusing on SGLT2 inhibitors. The leader of SGLT2 inhibitor research is the United States. Recently, research on SGLT2 inhibitors has focused on clinical studies, related mechanisms, and therapy. Future studies on SGLT2 inhibitors are expected to continue to focus on heart failure and diabetic cardiomyopathy, as well as delve further into the relevant molecular mechanisms, especially those related to fibrosis and AMPK. Revealing their relationship may be a new research frontier.

## Data Availability

The original contributions presented in the study are included in the article, further inquiries can be directed to the corresponding author.
